# Psychosocial burden of sickle cell disease on the family, Nigeria

**DOI:** 10.4102/phcfm.v4i1.380

**Published:** 2012-04-24

**Authors:** Samuel A. Adegoke, Emmanuel A. Kuteyi

**Affiliations:** 1 Department of Paediatrics and Child Health, Obafemi Awolowo University, Ile-Ife, Nigeria; 2 Department of Community Health, Obafemi Awolowo University, Ile-Ife, Nigeria

## Abstract

**Background:**

Sickle Cell Disease (SCD), the most common genetic disorder amongst Black people, poses a significant psychosocial burden on the sufferers, the caregivers and their families.

**Objective and methods:**

From 01 January 2011 to 30 September 2011 the caregivers of children with SCD treated at the Paediatric Haematology Clinic of the University Teaching Hospital in Ado-Ekiti, Nigeria, were included in a study, using a structured questionnaire and a validated, culture-relevant disease burden interview to assess the psychosocial burden of SCD on these caregivers. Three main objective psychosocial domains and some subjective domains, including the caregivers’ coping ability were assessed.

**Results:**

A total of 225 caregivers, consisting of 202 mothers (89.8%), 15 grandmothers (6.7%) and 8 fathers (3.6%) were studied. In 53.3% of the families, the care of children with SCD adversely affected the provision of their basic needs, with 73.3% of the caregivers stating that time spent caring for the child made them lose income or financial benefits; 19.6% of the caregivers had to take out loans to meet the expenditure of the patient's illness. Caring for children with SCD reportedly made 42.2% of the caregivers neglect other family members. In addition, 14.2% of the families experienced moderate to severe disruption in their day-to-day interactions within the family to the extent that 12.4% frequently quarrelled due to the child's illness.

**Conclusion:**

Caregivers are faced with enormous financial, interpersonal and psychological problems. Social support should be available to alleviate caregivers’ and/or family members’ burdens.

## Abstrait

**Le poids psychosocial de la drépanocytose sur la famille, Nigeria**

**Contexte:**

La drépanocytose, le trouble génétique le plus commun dans la population noire, constitue un poids psychosocial significatif pour les malades, les aides soignant et leurs familles.

**Objectif et méthodes:**

Du 1er Janvier au 30 Septembre 2011, les aides soignant d'enfants atteints de drépanocytose traités dans la clinique d'hématologie pédiatrique de l'hôpital universitaire d'Ado-Ekiti, au Nigeria, ont participé à une étude, réalisée à l'aide d'un questionnaire structuré et d'un entretien sur le poids de la maladie validé et pertinent en termes de culture afin d’évaluer le poids psychosocial de la drépanocytose sur ces aides soignant. Les trois principaux domaines psychosociaux objectifs et subjectifs, et la capacité des aides soignant à faire face ont été évalués.

**Résultats:**

Un total de 255 aides soignant, consistant en 202 mères (89.8%), 15 grands-mères (6.7%) et 8 pères (3.6%) ont participé à l’étude. Dans 53.3% des familles, la prise en charge des enfants atteints de drépanocytose affectait défavorablement la satisfaction de leurs besoins de base, 73.3% des aidants ayant déclaré que le temps consacré à s'occuper de l'enfant leur faisait perdre des revenus ou des avantages financiers; 19.6% des aides soignant ont dû contracter des emprunts pour pouvoir faire face aux dépenses liées à la maladie du patient. La prise en charge des enfants atteints de drépanocytose aurait poussé 42.2% des aides soignant à négliger les autres membres de la famille. De plus, 14.2% des familles ont connu une perturbation grave de leurs relations quotidiennes au sein de la famille, au point que 12.4% se disputaient fréquemment en raison de la maladie de l'enfant.

**Conclusion:**

Les aides soignant font face à d’énormes problèmes financiers, interpersonnels et psychologiques. Un soutien social devrait être proposé afin d'alléger le poids supporté par les aides soignant et/ou les membres de la famille.

## Introduction

Sickle Cell Disease (SCD), the most common genetic disorder amongst Black people and one of the major chronic non-communicable diseases (NCDS) affecting children, poses a significant psychosocial burden, not only on the sufferers but also on the caregivers and their families.^1^ The impact on the family is worse in developing countries such as Nigeria because of inadequate social welfare and health care services.^2^ Considering that the overall health of these patients depend on the quality of life and psychological preparedness of the caregivers, an assessment of the burden of the disease on these caregivers and family members is desirable.

Within the family micro-environment, children with SCD need optimal family support, understanding and care, particularly in terms of providing adequate nutrition and health care delivery so as to achieve an optimum and steady state of health. Such favourable family environment has been shown to be a good prognostic index.^1, 3, 4^

SCD afflicts up to 100 million people worldwide, predominantly amongst Black people in Africa, Europe and the America, Arabian people, and those of Asian ancestry.^4^ In Nigeria, it is estimated that about 150 000 children are born with sickle cell anaemia annually, with a prevalence of 20–30 per 1000 live births whilst that of haemoglobin SC is approximately 0.7%.^5^ The life expectancy of patients with SCD is quite variable. Some pass away at an early age whilst others have a virtually unrecognised condition and are able to live active lives, but only a few live until an advanced age.^5^

The level of support given to SCD children may reflect the dynamics of intra-family relationships and the emotional state of family members, especially in terms of the mothers of these children.^3, 4^ Despite the importance of a conducive home environment to the care of the SCD child, studies on the impact of sickle cell disease on the caregivers and family of the affected children are few, and therefore, attention to the psychosocial problems of mothers and/or caregivers of these children has been inadequate. This study therefore sets out to assess the psychosocial burden of sickle cell disease on the caregivers and families of the affected children treated at the University Teaching Hospital (UTH) in Ado-Ekiti, Nigeria, to determine the psychosocial needs of carers and affected family members.

### Setting

The study was done in an out-patient clinic setting of a tertiary hospital.

#### Key focus

Families of children with SCD are usually overwhelmed by the high level of burden of care in many parts of Africa.

#### Objectives and significance

SCD poses a lot of psychosocial burden not only on the sufferers, but also on the caregivers and their families. Assessment of the burden of the disease on the caregivers will therefore help in advocating necessary psychosocial supports needed by the affected families.

## Ethical considerations

Informed consent was taken from the caregivers involved in this study; those individuals who did not give consent were excluded from the study. In addition, ethical clearance was obtained from the Ethics and Research Committee of the hospital.

## Method

### Materials

A structured questionnaire was used to obtain information on the socio-demographic characteristics of the caregivers, such as the age, sex, social class (using the method recommended by Oyedeji^6^), number of children in the family, and the type of family setting. Other data included the biodata of the child, duration of care, frequency of hospitalisation, blood transfusion, and significant bone pain episodes since diagnosis. For the purpose of this study, a significant bone pain episode (or vaso-occlusive crisis) is defined as an acutely painful event requiring treatment at a healthcare facility or at home with either:^1, 4^

- parenteral or an equianalgesic dose of oral narcotics
- parenteral or an equianalgesic dose of oral non-steroidal anti-inflammatory drugs.

Sickle Cell Disease Burden Interview (SCDBI) was initially validated by Ohaeri and Shokunbi^7^ and found to be relevant to Nigerian culture. It was used to assess the psychosocial burden of the illness on caregivers and families and assessed three main objective psychosocial domains namely the financial burden of the disease, the disruption of family interactions, and the disruption of routine family activities. In addition, it assessed some subjective psychosocial burdens such as the caregiver's feelings (e.g. depression, sorrow, anger and/or stigmatisation) towards the child and the ability of the family to cope with the disease.

The SCDBI contained a total of 16 questions: three each on family finances and interactions, and five each on routine family activity and parental coping ability. Each question has a score ranging from 0 to 3. A score of 0 was given when the stressful event never occurred, 1 point was given when it occurred occasionally or had an insignificant impact on the family, 2 points were given when the stressful event occurred frequently or had a moderate impact on the family, and 3 points were given when it occurred regularly or had a severe impact on the family. The scores were then added for each of the psychosocial burden domains and the total score categorised and interpreted as follows:

- For impact on family finances and family interactions, total score of 0 as no impact; 1–3 as insignificant impact; 4–6 as moderate impact; and scores between 7 and 9 as severe impacts.
- For impact on routine family activity and parental coping ability, total score of 0 as no impact; 1–5 as insignificant impact; 6–10 as moderate impact; and scores between 11 and 15 as severe impact.

Data were analysed with SPSS 17.0. Frequencies, proportions, means, a standard deviation (s.d.) and a median were determined as appropriate.

### Context of the study

The study was carried out at the Paediatric Haematology Outpatient Clinic of the University Teaching Hospital (UTH) in Ado-Ekiti, Ekiti State, Nigeria. The hospital is a tertiary health institution and is the main referral facility providing both general and specialist paediatric care for many communities of south-western Nigeria.

### Design

This descriptive cross-sectional study was carried out over a period of 9 months (between January 2011 to September 2011). All the caregivers of children with SCD who were treated at the Paediatric Haematology Clinic of the hospital during the study period were assessed. To qualify for admission into the study, a caregiver must satisfy the following criteria:

- Caregivers must have attended the Paediatric Haematology Clinic at least 3 times prior to admission to ensure a reasonable period of interaction.
- The child must have been living with the caregiver for at least 1 year prior to the interview.
- The child was in a steady state (i.e. free from crisis in terms of bone pain and/or symptomatic anaemia, infection or any other illness) on the day the caregiver was interviewed.

## Results

### Age and sex distribution of the caregivers of children with Sickle Cell Disease

A total of 225 caregivers of children with SCD were assessed during the study period. They consisted of 202 mothers (89.8%), 15 grandmothers (6.7%) and 8 fathers (3.6%). Their ages ranged from 24 to 60 years with a mean (s.d.) of 41.2 (9.3) years. None of the caregivers was a teenager, 24 caregivers (10.7%) were in the age range of 20–29 years, 65 caregivers (28.9%) were in the age range of 30–39 years, 79 (35.1%) were in the age range of 40–49 years, and 57 caregivers (25.3%) were 50 years or older.

#### Characteristics of the children with Scikle Cell Disease

The mean age (s.d.) of the children with SCD was 7.2 (4.0) years, with a range of 2–17 years. Ninety-eight of the children (43.6%) were younger than 6 years, 67 (29.8%) were aged 6–10 years and 60 children (26.7%) were older than 10 years. One hundred and twenty-two children (54.2%) were male with a male to female ratio of 1.2:1. The majority of the children, namely 196 (87.1%), had sickle cell anaemia (haemoglobin SS), and 29 (12.9%) children had sickle cell haemoglobin C (haemoglobin SC).

#### Level of education and occupation of the caregivers

About a half of the caregivers (114, 50.7%) completed tertiary education, 82 caregivers (36.4%) completed secondary school education, 16 caregivers (7.1%) had primary school education and 13 caregivers (5.8%) had no formal education. One hundred and thirty (57.8%) of the caregivers were petty traders, artisans or full-time house-wives whilst the remaining 95 caregivers (42.2%) worked as public servants.

**Socio-economic status of the caregivers:** Whilst the majority of the caregivers (193, 85.8%) were from the lower social classes (III–V), only 32 caregivers (14.2%) were from the higher social classes (I and II). The average monthly income of the caregivers ranged from $10 to $470, with 114 caregivers (50.7%) earning less than $120 monthly, which is the national minimum wage in Nigeria.

#### Marital status and the family structure of the caregivers

One hundred and fifty two caregivers (67.6%) were from a monogamous family setting. In addition, whilst the majority of the caregivers (192, 85.3%) were married and living with their spouses, 6 caregivers (2.7%) were widowed, 24 (10.7%) of the parents were separated or had never been married, and 3 (1.3%) were divorced. Amongst most of the families, 155 caregivers (68.9%) had three or more children.

#### Clinical burden parameter

The duration of care of the children with SCD since diagnosis ranged from 4 to 148 months, with a mean (s.d.) of 47.0 (40.2) months. The median duration of care was 30 months. Whilst 92 (40.9%) of the SCD children had been admitted to hospital on at least three occasions as a result of SCD, 16 children (7.1%) had never been admitted before. Similarly, 105 children (46.7%) had been transfused at least twice for SCD-related anaemia and 146 children (64.9%) were said to have had significant bone pains on three or more occasions since diagnosis. Table 1 shows the detailed frequency distribution of clinical burden of sickle cell disease.

**TABLE 1:** The frequency distribution of clinical burden of sickle cell disease.

Indices of clinical burden of Sickle Cell Disease	Children	

	*n*	%
**Duration of care since diagnosis**		
≤ 6 months	22	9.8
> 6 months	203	90.2
**Previous hospitalisation (range = 0–24)**		
No hospitalisation	16	7.1
Once	32	14.2
Twice	85	37.8
> twice	92	40.9
**Previous blood transfusion (range = 0–10)**		
None	67	29.8
Once	53	23.6
Twice	70	31.1
> twice	35	15.6
**Significant bone pain episodes**		
None	13	5.8
One	41	18.2
Twice	25	11.1
> twice	146	64.9

*n*, Given as means of number.

#### Financial burden of Sickle Cell Disease on the caregivers

Table 2 shows the influence of SCD on the finances of caregivers, and indicates that 165 caregivers (73.3%) stated that time spent caring for the child made them lose income or financial benefits. Similarly, 120 caregivers (53.3%) reported that the expenses of the child's illness adversely affected the provision of their family's basic needs such as food, clothing and shelter and 44 caregivers (19.6%) had to take out a loan to meet the expenditure of the patient's illness.

**TABLE 2:** The impact of Sickle Cell Disease on the finances of the caregivers.

Indices of financial burden	Never occurred		Occurred sometimes		Occurred frequently or always	
		
	*n*	%	*n*	%	*n*	%
Lose income or financial benefits due to time spent caring for the child.	60	26.7	124	55.1	41	18.2
Took out a loan to meet expenditure of the child's illness.	181	80.4	29	12.9	15	6.7
Expenses of child's illness adversely affected the family's basic needs.	105	46.7	101	44.9	19	8.4

*n*, Number of caregivers.

#### Financial burden score

Using a total score of ‘0’, ‘1–3’, ‘4–6’ and ‘7–9’ for no financial impact, insignificant impact, moderate impact and severe impact respectively, 41 caregivers (18.2%) claimed that caring for a child with SCD did not have any effect on their finances. One hundred and forty-nine caregivers (66.2%) claimed that the effect on their finances was insignificant, 25 caregivers (11.1%) stated that the financial impact was moderate whilst the remaining 9 caregivers (4.0%) stated that the financial impact was severe.

#### Burden of Sickle Cell Disease on routine family activities

Table 3 shows the impact of SCD on routine family activities. Ninety-five (42.2%) caregivers stated that caring for their SCD children made them neglect other family members. Two hundred caregivers (88.9%) reported that the illness made it difficult for their children to attend school. Similarly, in 15 families (6.7%), SCD frequently or always prevented the affected child from assisting in household chores, whilst in 11 families (4.9%), the illness frequently made it difficult for other members of the family to engage in gainful activities such as going to the market or church. The child's behaviour or demands in terms of the child's health-care frequently disturbed recreational activities in 10 homes (4.4%).

**TABLE 3:** The impact of Sickle Cell Disease on routine family activities.

Indices of disruption of routine family activities	Never occurred		Occurred sometimes		Occurred frequently or always	
		
	*n*	%	*n*	%	*n*	%
Caring for the child caused neglect of other family members.	130	57.8	89	39.6	6	2.6
Illness made it difficult for the child to assist in household chores.	67	29.8	143	63.6	15	6.7
Illness made it difficult for the child to attend school.	25	11.1	181	80.4	19	8.4
Child's illness disturbs activities at home (e.g. recreational activities).	129	57.3	86	38.2	10	4.4
Caring for the child made it difficult for the caregiver to engage in other gainful activities.	149	66.2	65	28.9	11	4.9

*n*, Number of caregivers.

#### Family routine burden score

Although none of the caregivers of SCD children experienced a severe disruption in routine family activities (i.e. total score 11–15), 16 caregivers (7.1%) experienced moderate disruption (i.e. total score 6–10) and 206 caregivers (91.6%) experienced insignificant disruption (i.e. total score 1–5) in routine family activities. Only 3 caregivers (1.3%) had a total score of ‘0’, indicating that caring for children with SCD did not have an impact on their routine family activities.

#### Burden of Sickle Cell Disease on family interaction

Table 4 shows the effect of SCD on family interactions. Sixty-seven caregivers (29.8%) stated that their children's illness caused a general atmosphere of tension or hostility in their homes. Twenty-eight caregivers (12.4%) quarrel frequently or regularly with their spouses because of the child's illness, whilst 41 caregivers (18.2%) had experienced marital disharmony.

**TABLE 4:** The impact of Sickle Cell Disease on the family interactions.

Indices of disruption of family interactions	Never occurred		Occurred sometimes		Occurred frequently or always	
		
	*n*	%	*n*	%	*n*	%
Child's illness causing a general atmosphere of tension or hostility in the home.	158	70.2	48	21.3	19	8.4
Child's illness or care causing disagreement or quarrels amongst family members.	165	73.3	32	14.2	28	12.4
Child's illness causing marital disharmony, (e.g. threat of separation or divorce).	184	81.8	25	11.1	16	7.1

*n*, Number of caregivers.

#### Family disharmony score

In 13 of the families (5.8%), caring for children with SCD had severely disrupted family interactions (i.e. total score of 7–9). Whilst 19 families (8.4%) had experienced moderate disruption in family interaction (i.e. total score of 4–6), a further 48 families (21.3%) experienced insignificant disruption (i.e. total score of 1–3). The majority of the families (145, 64.4%), however, did not experience any disruption in their family interaction (i.e. total score of 0).

#### Burden of Sickle Cell Disease on the caregivers’ coping ability

Seventy-six caregivers (33.8%) had difficulty coping with the care of their children. Most caregivers (196, 87.1%), however, had no difficulty accepting the responsibility of caring for them. The majority of the caregivers (190, 84.4%) felt sorrowful or depressed about their child's illness, 70 caregivers (31.1%) frequently or always felt angry with themselves or with the child because of his or her illness, and 61 caregivers (27.1%) frequently or always felt stigmatised. Table 5 shows the impact of SCD on caregivers’ coping ability and their feelings towards the affected children.

**TABLE 5:** Impact of Sickle Cell Disease on caregivers’ coping ability and their feelings towards the affected children.

Indices of caregivers’ coping ability and feelings towards the affected child	Never occurred		Occurred sometimes		Occurred frequently or always	
		
	*n*	%	*n*	%	*n*	%
Difficulty coping with the child's illness.	149	66.2	63	28.0	13	5.8
Difficulty accepting responsibility to care for the child.	196	87.1	25	11.1	4	1.8
Feeling sorrowful or depressed about the child's illness.	35	15.6	98	43.6	92	40.8
Feeling angry with self or the child because of his or her illness.	76	33.8	79	35.1	70	31.1
Feeling stigmatised because of the child's illness.	120	53.3	44	19.6	61	27.1

*n*, Number of caregivers.

## Discussion

The SCD sufferers, as well as their caregivers, are faced with several challenges such as daily use of routine drugs, recurrent or frequent illnesses, the need for blood transfusion, regular clinic attendance and hospitalisation. Hence, parents or caregivers of these children tend to have worse health-related quality of life, compared to those without SCD children which impacts negatively on their behaviour and self-esteem.^2, 8^

In the present study, the socio-demographic characteristics of SCD caregivers were similar to those of other studies in the developing countries such as Nigeria.^7, 9^ Whilst only about half of the caregivers completed tertiary education, approximately 13% either did not have any formal education or just completed primary school education. In addition, the socio-economic stratification of the caregivers was similar to that of parents of children with other illnesses in south-western Nigeria^7, 10, 11^ There is therefore no reason to suggest that the participants involved in this study differed in socio-demographic characteristics from the general population in our environment other than the illness variable.

In this study, the rates of bone pain episodes and hospitalisation amongst the SCD children were high. About 6% of the children had never experienced significant bone pain episodes. Only 7% of the children had never been admitted before. This supports the hypothesis that bone pain episodes account for the reasons why SCD children are admitted in most cases.^7^ Hence, if bone pain episodes can be controlled, the social as well as the psychological burdens of the disease may be ameliorated.

The financial burden of SCD on the caregivers and their families is high in this study. More than half of the caregivers reported that the expenses of the child's illness adversely affected the family's basic needs such as food and house rent. This is not surprising considering the rising trend of inflation in this period of global economic recession and socio-political instability. In Nigeria, like many other developing countries, national programmes on health insurance and social welfare systems are absent, making caring for a child with chronic illnesses such as SCD a great financial burden. Furthermore, about 70% of the caregivers in this study lost income or financial benefits due to time spent caring for their children. In Nigeria, the predominant form of health-care financing is out-of-pocket. As observed previously, job loss, underemployment and/or unemployment arising from time spent caring for a child with SCD, will significantly contribute to the financial burden experienced by caregivers and their family.^2^

In the present study, about 40% of the caregivers sometimes or frequently neglected other members of the family because of the demands caused by the child's illness. It is known that the way parents relate with their ill children and the feeling of neglect this generates in other siblings is a major factor in family dysfunction.^9^ This neglect especially when experienced too frequently has been described as a risk factor in the psychopathology of psychosocial problems in chronic physical illness.^12^ Frequent school absenteeism as a result of recurrent crises and suboptimal health is another major problem of SCD children, as shown in this study. It is important, therefore, that adequate and concrete educational plans should be developed for them.

Family life and interactions have been described as significant areas of social life for SCD patients and their families.^7^ In this study, at least 70% of the caregivers reported intact family relationships in the three indices assessed. This is similar to findings by Ohaeri^7^ and Adeodu^13^. The reason for this may be because of the ameliorating effect of the extended family structure, culture and religion. In Nigeria, beliefs are influenced by cultural and religious values, which in turn influence health behaviour and coping strategies.^14^

Unlike previous research conducted in Nigeria^7, 15^ which reported that families of individuals with SCD deny evidence of stigmatisation, about half of the caregivers in this study reported the existence of stigmatisation. However, this is consistent with findings in developed countries such as the United Kingdom (UK) and the United States of America (USA) where stigmatisation has been described as a prominent social problem in mothers of Afro-Caribbean children with SCD who are living outside Africa.^14^

Depression was also experienced by the caregivers interviewed. This is consistent with a study on psychosocial and family functioning in children with SCD and their mothers in Atlanta, Georgia, where they found that sickle cell patients and their caregivers experienced more depressive symptoms than the controls.^16^ Similar studies in developing countries such as Nigeria also agree with this high level of depression amongst caregivers of chronic illness.^7, 9, 15^

In this study, caregivers coped differently. Some of them were able to cope relatively well, especially in routine family activities. However, others coped inadequately, resulting in feelings of depression, sorrow and anger towards themselves and the affected child. This variability in the coping ability of the caregivers may not necessarily be a consequence of the severity of the clinical condition of the child, but several other factors such as the socio-demographic characteristics of the caregivers and the social or family support available to them.

The findings of this study suggest that caregivers of SCD children experience considerable psychosocial impairment from their children's illness. Hence, more attention is needed in this regard, since psychosocial maladjustment is known to aggravate problems of SCD children.^9, 17, 18^ Social welfare programmes such as national health insurance for children with chronic illnesses will definitely alleviate the associated financial burden. For instance, the caregivers of SCD children in western countries such as the UK have the opportunity to benefit from health insurance and hence, they tend to report lower financial burdens compared with their counterparts in most parts of the developing world.^18^ Other social welfare programmes and self-help group programmes are also vital in relieving the psychosocial burden of disease and consequently, improving the quality of care for the SCD patients. Presently, in Nigeria, the National Sickle Cell Association and Sickle Cell Disease clubs are based in a few tertiary health institutions with insufficient community impact.^7, 13^ These social organisations should be encouraged so that SCD children and their caregivers can share their feelings and counsel one another. Other measures to reduce the impact of SCD on caregivers include limitation of family size to reduce the risk of mothers from having additional SCD children. In addition, there should be promotion of neonatal screening, genetic counselling and comprehensive public health education. The latter must aim at increasing community awareness on the burden and prevention of the disease. Routine haemoglobin genotype determination for adolescents before entering into marital relationships is vital. This has been noted to offer a pragmatic approach in reducing the high prevalence of the sickle cell gene and the attendant problems in Nigeria.^13^

### Limitations of the study

A limitation to this study was our reliance on the caregivers to speak on behalf of their families, because it is difficult to objectively quantitate emotional stress and disturbed intra-family ties. However, the careful selection of these caregivers ameliorated the possible effect of this potential source of bias.

### Recommendations

Government should strengthen the existing national health insurance as well as subsidising the cost of SCD care to alleviate the huge financial burden on the family. In addition, regular psychological support should be available to alleviate caregivers’ and/or family members’ burdens.

## Conclusion

In conclusion, this study has shown that the caregivers and/or families of children with SCD experience a significant psychosocial burden. Hence, clinicians and policy makers should provide the necessary psychological care and support to these individuals.
